# Facebook Feeds and Child Feeding: A Qualitative Study of Thai Mothers in Online Child Feeding Support Groups

**DOI:** 10.3390/ijerph19105882

**Published:** 2022-05-12

**Authors:** Abhirat Supthanasup, Cathy Banwell, Matthew Kelly

**Affiliations:** 1 National Centre for Epidemiology and Population Health, Australian National University, Acton, Canberra 2601, Australia; cathy.banwell@anu.edu.au (C.B.); matthew.kelly@anu.edu.au (M.K.); 2 School of Human Ecology, Sukhothai Thammathirat Open University, Nonthaburi 11120, Thailand

**Keywords:** social media, Facebook, infants and young child feeding, qualitative, Thai

## Abstract

Mothers have traditionally sought child feeding information from social connections. While mothers are heavily engaged on social media and value peer support in online communities, very little is known about how they use online communities for information about child feeding practices after exclusive breastfeeding cessation. This study explores mothers’ perceptions of joining Facebook child feeding support groups. Individual semi-structured interviews with ten Thai mothers were conducted. The transcribed interviews were analyzed using a phenomenological hermeneutical approach. Our findings highlighted that Thai mothers participated in Facebook child feeding support groups in a deliberate effort to reduce their uncertainty by normalizing the process through accessing the shared experiences of others. One of their intentions was to seek menu recipes based on favorable psychosocial and environmental factors. Implications for using social media in health promotion and communication include the importance of building appropriate common practices through social collaboration and interactivity to supplement traditional knowledge and attitudes.

## 1. Introduction

Social media has become deeply embedded in modern society, including in developing countries. In Thailand in 2015, the number of active social media users was just 58% of the total population [1]. There has been a significant rise to three-fourths of the total population in 2020 [2]. The bombardment of social media has triggered a shift in health information seeking behavior toward online society. A mounting body of evidence suggested that mothers are heavy users of social media [3,4,5], especially mothers of young children [6]. According to a survey in Thailand, around 70% of respondents, especially millennial mothers with their first child, participated in social media that provided information about children’s diets [7]. Among all social media services, Facebook is gaining increased attention as a platform used by parents [5,8]. As social media is specifically designed to promote interaction-communication [9], the increasing number of mothers on social media produces a new form of community-based communication which called social media groups for parents. For example, a previous study found that there were nearly forty Facebook peer-support groups in young children’s diets in Thai language, of which the most popular group had a membership of around 250,000 [10].

The transition to parenthood comes with many challenges, leading parents to seek social support from their connections [11]. Emerging social media groups have facilitated the fluidity of parenting conversations into virtual space, where mothers can reach and contribute anywhere, anytime. This insertion of the social media group in the 21st century may serve as a new mechanism for gaining social support for modern parents. Previous studies revealed that social media groups have already contributed to informational and emotional support for parents [10,12]. Additionally, a Pew research study found that 74% of parents who engaged with social media get support from their social network [13]. However, social media use and making social comparisons online may be associated with adverse outcomes in the context of highly contested ideas about mothering [14]. Even though previous studies have pointed out that social media has pros and cons for parents using online platforms, social media is a preferred source for child feeding information over the government among millennial Thai mothers with their first child [7]. In Thailand, the Maternal and Child Health (MCH) handbook is key document in the primary health care approach to promote maternal and child health. As the Universal Health Coverage (UHC) scheme has been implemented to increase the accessibility of health care service, mothers and child have free access to services, including continuum of antenatal care. Therefore, the MCH handbook, developed by the Ministry of Public Health, has been disseminated to pregnant women who receive antenatal care throughout the country [15]. This MCH handbook covers various topics, including complementary feeding guidelines. However, a study in 2015 found that the number of Thai parents using the MCH handbook was low [16]. Very little is known about why parents have shifted their information-seeking behavior from traditional sources to social media. As the growing influence of social media adds an additional layer of complexity to public health information, studies are needed to ascertain how these platforms function related to traditional sources.

Maternal child feeding is among the parental practices which are complicated by influences in the social environment. Traditionally, mothers have employed cultural capital, displayed as social norms and beliefs, to dictate what and how to feed their children [17,18], some of which can also become barrier to following feeding recommendations [19]. In the 21st century, as parents are increasingly seeking advice on social media, social media groups are likely to facilitate social learning in ways that other online contexts do not, by establishing normative practices within online communities. The literature in this area proposes that content contribution and observation, the most common online activity, may facilitate observational beliefs and behaviors [20]. Preliminary work investigating so-called mommy blogs concluded that most blog posts contained information on child feeding beliefs and behaviors which may influence readers’ child feeding practices [21]. More recent studies explored online peers’ conversations in Facebook groups related to child feeding [10,22]. These works revealed that recipes were a common topic on these Facebook groups. A study that analyzed recipe posts shared in Facebook groups found that these disseminated food choices that were seen as appropriate [23]. For example, ideas around food allergy awareness resulted in animal-sourced foods being rarely used in shared recipes aimed at younger children. These circulating ideas on social media could explain why more than half of Thai children still have challenges meeting minimum dietary diversity, especially at the early complementary feeding stage [24]. Interestingly, user-generated content has been demonstrated to be more popular and effective than professional-generated content [25]. A study investigating cross-cultural differences in online health information seeking found that Asian populations are more likely to trust and use experienced-based information from lay people [26]. Thai millennial mothers have also been found to be highly engaged with social media and tend to trust shared information [7]. Thus, the explosion of online content has the potential to shape parental feeding practices in modern Thai society. However, further study is required to address how online interactions and content exposure influence parents’ child feeding attitudes and practices and how they consume shared recipe content.

### Rationale and Aim

Parents have historically mostly sought guidance and support from a small circle of family, friends, and health institutions. However, modern parents are increasingly turning to social media for advice. Social media, especially Facebook, has commonly been mentioned as online sources for pregnant women and mothers of young children [5,27]. As studies on child feeding after exclusive breastfeeding cessation have concentrated on parents’ characteristics and discussion content, interview studies are needed to expand and deepen the current knowledge. This is important, as Thai millennials are digital natives and could be considered the first group of mothers to go online for information. Qualitative data can offer rich insights into why and how millennial mothers seek child feeding advice on online support groups, which can help guide better-designed and better-implemented child feeding programs. Therefore, the aim of this study was (1) to explore why Thai millennial mothers join Facebook child feeding support groups (2) to understand how they experience online peer groups and (3) to understand how they received and interpreted content and its impact on child feeding decisions.

## 2. Materials and Methods

### 2.1. Study Design

Phenomenology, the philosophical paradigm underpinning this qualitative research encourages researchers to explore and understand phenomena from personal experience [28,29]. This approach was chosen as it is consistent with our aim to explore Thai parents’ experiences and perceptions of joining Facebook child feeding support groups.

### 2.2. Participants and Recruitment

Participants were recruited through purposive sampling. Because social media use for parenting purposes is correlated with gender and generation, the participant candidates were selected because they were millennial mothers, born between 1981 and 2000. Recruitment was conducted among 379 parents who participated in the authors’ previous survey, which assessed the prevalence and characteristics of Thai parents’ participation in social networking sites about child feeding [7], and who agreed to further contact. This qualitative study explores in-depth the findings from the more generalizable survey by recruiting participants who were already users of social media for information about child feeding. Participant candidates were contacted initially by phone or email invitations. If they were interested in participating, a consent form was sent by email and supplemented with other online communication they preferred. They were then telephoned, emailed or texted to arrange a time and method for the individual interview. Recruitment continued until data saturation was reached after analysis of the first seven interviews, and when no new codes or themes were revealed. However, an additional three interviews were conducted to confirm that no new information was gained.

### 2.3. Characteristics of Participants

All participants (*n* = 10) were Thai millennial mothers, and their mean age was 33 years. Two had more than one child at the time of the interviews. They all had education levels at bachelor or higher university degrees. Of the 10 participants, 9 worked full-time outside home. They were all married or living with a partner. They reported firstly participating in any Facebook child feeding support group while their child was around 5–6 months old, during their first child feeding transition period from exclusive breastfeeding to complementary feeding food. They all reported spending at least an hour daily on social media, and 15–30 min per visit on the Facebook child feeding support group.

### 2.4. Data Collection

In-depth, semi-structured interviews were conducted during January 2021 and April 2021, using participants’ preferred technology, mostly by voice call on the Line application. The interview guide (Appendix A) was constructed based on authors’ previous studies [7,10,23]. All interviews were conducted in a private location where the conversation could not be seen or heard by others. Eight participants were interviewed in a private room. Others were interviewed in their car. The interviews last between 15–40 min (mean = 23 min). The audio was recorded and transcribed verbatim into separate Microsoft Word documents; all identifiable data were modified.

### 2.5. Data Analysis

A phenomenological hermeneutical method was used in the interview data analysis. Following Lindseth and Norberg [30], the method comprises three steps: naive reading, structural analysis, and comprehensive understanding. An initial reading of interview data, transcript by transcript, provided a naive understanding of the whole data set which guided the structural analysis. During the next stage, the text was divided into portions of texts called meaning units, similar to coded text. The meaning units were then condensed and abstracted to build subthemes and themes. The third step aimed to gain a comprehensive understanding based on this work and the authors’ preunderstanding. The first author performed all these steps and then discussed the selected excerpts and emergent themes with other authors until the consensus was reached.

## 3. Results

### 3.1. Why Are Mothers Going Online?

#### 3.1.1. Effort to Reduce Uncertainty during Feeding Transition by Learning What Other Mothers Do

The transition from exclusive breastfeeding to complementary food raises mothers’ uncertainty about what and how to feed their child. This uncertainty, in turn, encourages them to gather information. In practice, mothers have historically sought out parenting support through their numerous social connections. As social media has emerged, a response to this uncertainty has shifted beyond the typical network of influence to a virtual community. A 34-year-old mother described herself as a group member because of a lack of confidence in starting complementary feeding:

“*I have to start feeding my child, but I don’t know what or how. I don’t normally cook. So that’s why I turn to Facebook groups on child feeding for parents… I have been using social media and joining many Facebook groups. I can feel that there might be Facebook groups on child feeding for parents. Then I searched and found them.*”

(Interview 2)

Another mother explained that being a first-time mother, she finds this overwhelming. As a result of being such an inexperienced mother, she turned to participate in a Facebook group on child feeding:

“*I’m a first-time mother with no experience. I would like to know what other mothers are doing for their child’s food. Just in case, I can use their experiences as a guideline. Then I joined a Facebook group.*”

(Interview 1)

Some mothers mentioned that they more frequently visited these groups when their children were in the feeding transition period, not only to initiate new food but also to modify the family diets. The uncertain transition to a new food regimen compels mothers to seek out what other parents are feeding their children at the same age. As a 34-year-old mother from north-eastern Thailand said:

“*I frequently visited the groups when my child was in a transition period. I meant when (s)he was 6–7 month-old and 1–2-year-old. Oh! And when (s)he was a picky eater. I was there to see what others do for menu inspiration. It helped because if I have no idea what to feed my child, I will offer him(her) the same menu. (S)He will get the same nutrients.*”

(Interview 1)

#### 3.1.2. Seek out Alternative or Complementary Support to Satisfy Their Practical Needs

In the meantime, some mothers in this study were dissatisfied with the national guideline and sought peer support in Facebook groups as alternative or complementary information. Their needs and expectations were not met by the guideline. A mother from north Thailand explained that the guideline focuses on the amount of each food groups rather than instruction on how to make children’s meals by using the suggested food:

“*Mother and child health book provides a very broad guideline. It explains what I should offer my child in each food groups at tablespoon unit. But what should I do with these ingredients? Okay, it gives me an idea, such as half an egg, a teaspoon of oil, but what should I do next? What should I do with this egg and oil? I have no idea. But when I joined a Facebook group. Some mothers shared their recipes and gave me an instruction how to make it in detail. I do like seeing what others do step by step.*”

(Interview 6)

While another mother explained that:

“*The mother and child health book suggests that I should give my child egg yolk, liver, and vegetable. But it doesn’t tell me which vegetable. But once I joined a Facebook group, I have learned more about vegetables from the shared recipes.*”

(Interview 8)

Some parents viewed the official guideline and Facebook pages from healthcare professionals as theoretical knowledge that lacked practical application. They joined a peer support group to seek the missing practical information to help them make food for their child. As one mother in this study explained:

“*The information obtained from the pink book (maternal and child health book) is kind of theory. There is a table of guideline for complementary feeding. But actually, when it comes to raising children, it is not exactly the same as that sort of information. I need to learn from other mothers’ experiences rather than just following “one size fits all” information as in the guideline.*”

(Interview 1)

Another mother explained:

“*I also followed a Facebook page by a healthcare professional. I felt I am reading a textbook. Can you imagine? But I need something more than just a standard guideline. I need to learn in a practical way that I can implement in my situation. When I joined a peer group, I can feel like indulging in girl-talk gossip with friends. Can you imagine? It is more intimate. Being around many mothers motivated me to learn and apply to my context. I have learned from what other mothers did or are doing. It’s personal experiences on child feeding.*”

(Interview 2)

Parents in the 21st-century have moved their education to an online and social network. Social media offers a convenient way for parents to access needed information. Instead of reading official guidelines or parenting books, many mothers preferred to join Facebook groups to gain information, such as a sample menu for their children. They find it easier to reach their needs online than through parenting books. One mother explained she preferred to go online over traditional information because she can find information at her fingertips. She explained:

“*I have to accept that there is detailed information in the pink book. Such as how many spoons I should feed my child. But nowadays, I preferred to go online. I open Facebook app on my phone and enter the keyword to search for posts in a Facebook group. There is a function to search for posts that have the information I want. It is very convenient. But for the pink book, I keep it in the cupboard. I have spent more time opening the cupboard and search information in the book. I found that social media is easier and more convenient.*”

(Interview 5)

A 31-year-old mother from southern Thailand explicated her information-seeking behaviour in the digital age. She preferred going online over visiting a bookstore which made her life easier and more convenient:

“*Nowadays, it’s out of my mind to visit a bookstore to buy a parenting book on baby food cooking. Joining a Facebook group is more effortless. When I joined the group, I saw what others did. I can learn the process. For example, others will tell you how to start, how to do it, and what the child’s meal looks like. It’s easy to follow.*”

(Interview 3)

Some mothers valued advice from family members. However, they found this advice out of date. A 34-year-old mother from central Thailand explained:

“*My parents raised me, and I admired them. But I have my vision of raising my child. I didn’t follow them for giving my child orange juice or [name of commercial baby food]. They are high in sugar. And I didn’t give my child water after breastfeeding. I think that advice is not up to date with current knowledge.*”

(Interview 2)

A mother from north Thailand said:

“*I didn’t give my child only mashed banana and rice like my parents’ advice. Now there should be added eggs and meats.*”

(Interview 4)

### 3.2. How Do Mothers Find Being a Member of Facebook Group?

#### 3.2.1. Gain Self-Efficacy by Receiving Experiential Supports

Mothers in this study frequently said that they did not join Facebook groups to obtain theoretical knowledge but instead to gain experiential information and support. All mothers found being a member of the Facebook group was helpful as it provided a shared space in which to learn home cooked recipes from other mothers. The shared experiences from others increased their self-efficacy about child feeding practices. A 33-year-old mother, who previously described herself as lacking confidence in making child food, said:

“*I rarely cook, so I do not know much about vegetables. Once I joined the group, I have learned more about vegetables. Such as butternut, I do not know what it is. I found out that it is like a pumpkin, and it is delicious. Or beetroot, I have tried it once in my salad. But I have no idea what dish I can use as an ingredient. I have learned from the group that I can steam and mix it with other ingredients to make my child’s meal. I think I have gained more knowledge and skills.*”

(Interview 6)

While another mother commented:

“*I feel much more confident when I see others have worked through their child feeding practice. For example, I do not know how to make a sweet and sour stir-fry. I do not cook. So it was challenging for me more than others who have cooking skills. But when I saw what others do, it was not that hard.*”

(Interview 3)

Generally, participants felt that they received experiential support from others. Passing on their personal experiences to others played an important role as community members. They felt that their experiences could help in validating and reassuring others:

“*I have shared my experience on how to classify ivy gourd. I would like to help others to clarify their curiosity. I understand how others are stressed out about their child feeding. I have passed through, and I would like to help. I know infants and young children cannot have family food, am I right?*”

(Interviews 8)

#### 3.2.2. Knowing Others’ Feeding Problems Helps Parents to Feel Normal and Gives Them Emotional Support

Reading others’ conversations around their child feeding issues may help reduce stress. Some parents mentioned that observing others’ experiences dealing with children’s picky eating enabled them to know that their problems were normal relative to others. A mother from south Thailand simply explained:

“*When I visited the group and saw other parents shared their problems, I felt I am not alone. Such as a problem of child picky eating. Understanding of this is normal makes me feel more relaxed in raising my child.*”

(Interview 3)

### 3.3. How Do Mothers Consume Shared Recipe Contents?

Generally, participants in this study came to the Facebook groups on child feeding to seek menu inspiration. Some parents admitted that they follow common child feeding practices indicated in many shared recipe descriptions, such as introducing new foods with allergy awareness, and no sugar and salt added to meals for children under one-year-old. However, in this section, we concentrated on how mothers select menus. We found that psychosocial and environmental factors were related to this selection process.

#### 3.3.1. Visually Appealing Pictures of Shared Recipes

It is a common practice that online parents share their home cooked recipes with pictures. Visualizing shared recipes seem to heighten parents’ willingness to follow the shared recipes. Rather than text-based cooking instructions, some parents would follow the particular shared recipes when they see what the meal’s appearance looks like. Some parents described how and why these food pictures of shared recipes attract their attention:

“*Parents love taking pictures of their child’s meals. They posted the ingredients list, cooking instructions with these pictures. I am always looking for a lovely meal with colourful ingredients. I think an attractive meal would build up children’s appetite.*”

(Interview 5)

Another mother explained further that the appearance of food fed to children aged over one year old is more attractive:

“*I mostly look at shared recipes with beautiful pictures. I think the way how to plate your child’s meal would affect their appetite. I meant the colour and presentation. I know the nutritional values come first. But when my child reached one or two years old, I need something more creative. Seeing how others beautifully plating their child’s meal inspired me to follow them.*”

(Interview 8)

#### 3.3.2. Peer Endorsement

Instead of reading through just the original posts, we found that following others’ conversation under the thread gave a wider perspective. Some parents admitted that the endorsement of peers under the thread impacted whether they followed the shared experiences of the initial thread:

“*In addition to reading the initial contents, I read what other mothers are saying about the thread. I have been reading comments under the shared recipes thread. An avocado dish, for example. When other mothers, saying “my child likes having avocado”, I prefer to follow that recipe. It is like a menu recommendation at a restaurant.*”

(Interview10)

Some parents said that drawing on many different posts/groups was one of the strategies used in making a decision when they come to unfamiliar main ingredients. As one mother in this study explained:

“*Once I found the content that I am interested in, I always seeking additional information. I read through several posts from many Facebook groups before making a decision. I would like to know what others are saying about that particular issue or ingredient. I am seeking what is the reason others support these feeding practices or ingredient.*”

(Interview 9)

However, a few parents said that they were not engaging with comments under the thread. This mother of three sons from southern Thailand said:

“*I do not read comments, but I am interested in pictures of shared recipes. I think each kid has their own food preferences. For example, when the poster and other mothers saying “my child like this dish” or “my child always finish this menu”. But that is not a guarantee for my children.*”

(Interview 7)

#### 3.3.3. Food Availability and Accessibility

Food availability is an important factor when making a decision. In this study, some parents said the availability of ingredients in shared recipes tended to shift their decision. A mother from north Thailand said:

“*I always follow the shared recipes which I already have some ingredients in my kitchen.*”

(Interview 4)

In virtual communities, food availability and accessibility sometimes depend on informational support from others. When food ingredients in shared recipes was unfamiliar or not locally produced, there was informational support on where to buy them. A mother explained her intention to help others on ingredients seeking:

“*I have shared with others about where to buy avocado and butternut.*”

(Interview 10)

#### 3.3.4. Child Preference

The menu selection is not just dictated by food availability and accessibility and psychosocial influences. Child food preferences predominantly impact them. Most mothers in this study mentioned that their child’s food preferences always determine whether they adopt the shared recipes on Facebook groups. One mother who is not engaged with the crowd said:

“*I rarely follow the shared menu that got many likes. I always seek for menu based on my child’s preferences. This is my major criteria.*”

(Interviews 6)

Food allergies and intolerances also play this role in menu selection. A mother of a child with an egg allergy described her menu selection criteria:

“*Even the picture of food appearance attract my attention, I always seek menu without egg because my child can’t have it as a trigger of allergy.*”

(Interview 10)

However, some mothers described that they try introducing new foods, which they found in groups, and see how their child likes them. One mother explained her experience:

“*I tried to introduce my child to a preservative-free sausage when s(he) was five years old. I found this sausage shared on Facebook groups. But s(he) do not like it much. Then I stop cooking this menu.*”

(Interview 4)

## 4. Discussion

This qualitative study offers insights into the maternal perception of shifting to an online community. In a previous study, Thai parents reported that doctors, family members, books, the internet, and social media are more frequently used than government sources for child feeding information [7]. In this study, a lack of practical tips in government guidelines on healthy food for children and out-of-date advice from family members, contributed to modern mothers’ experiences of uncertainty about feeding their young child. Furthermore, books have been viewed as an inconvenient source of information and support. As a result, they move beyond a typical social network of female friends and relatives to the larger scale support through an online community. Similar to a previous study, mothers found information on the NHS as a one-size-fits-all approach and valued more specific practical information gained on social media breastfeeding support groups [12]. Online social networks are an emerging trend that provide opportunities to encourage peer support in various settings. Online peer support has been oriented toward patients with chronic conditions (e.g., cancer, diabetes) [31,32] and others intending to change a health-risk behavior (e.g., smoking cessation) [33]. These online communities offer experienced-based information and reciprocal emotional support. Our previous thematic content analysis also found that Thai Facebook support groups on children’s diets have become a venue for parents to seek informational and emotional support [10]. Being a member of a social media breastfeeding support group increased mothers’ self-efficacy [12]. This finding is in line with our present study that Thai mothers had gained self-efficacy in child feeding practices through receiving experiential support from online peers. A recent review revealed that mothers in online communities exchanged experience-based knowledge, which was perceived as credible and helpful to their specific situation [34]. Learning about others’ experiences can increase confidence [35]. Therefore, online support-seeking behavior seems to be a 21st century solution to reduce uncertainty and boost confidence around child feeding.

Parenting is socially constructed and linked to social support. Mothers have historically sought social support from family members and community during the transition to parenthood [11]. As revealed by a previous qualitative study, parents learned about child feeding practices from peers’, friends’, and family’s experiences by observation and discussion [36]. In online society, recent literature claimed that mothers turn to social media for parenting advice as they receive immediate support and tailored information [37]. Our findings further explained that Thai mothers deliberately participate in Facebook groups on child feeding to reduce their uncertainty about the feeding transition through a normalization process gained through accessing the multiple practices and experiences of others. This social process is facilitated through observing, contributing, and reinforcing within online communities. As facilitated by these social media functions, common child feeding practices in social media may evolve into social norms [20]. In our previous studies, we found that there were commonly shared practices around feeding age-appropriate food and seasoning, and food allergy awareness, which was disseminated within Thai Facebook groups in children’s diets [10,23]. In the interviews, some mothers explained why they had adopted them. As both uncertainty and perceived shared identity are drivers of norms [38], new group members may easily adopt these common group practices.

Among the plethora of content, mothers in this study focused on shared home-cooked recipes for infants and young children based on psychosocial and environmental factors. From a psychosocial perspective, visually appealing pictures of shared recipes and peer endorsement were mentioned as influences on whether mothers adopted shared recipes. This is consistent with a reasoned action approach which argues that intentions are determined by three psychosocial variables: attitude, normative influence, and self-efficacy [39]. Visually appealing pictures of shared recipes influenced mothers’ thoughts and feelings, which can be determinants of attitude, whereas, other members also enforce decision-making as peer endorsement. This process is associated with normative influence in online groups. While attitudes and normative influences are primarily drivers of menu selection, group support could be considered a self-efficacy construct. Mothers in this study perceived that they gained necessary skills and abilities to cook children’s diets. This finding is consistent with previous results that group support provided mothers with confidence [12]. Apart from psychosocial factors, food availability and accessibility and child food preferences influenced maternal decision-making about shared recipes. Psychosocial factors can be stimulated or interfered with by these environmental factors. Interestingly, in this study, peers discussing where to buy unfamiliar food ingredients can diminish food accessibility and availability barriers.

### 4.1. Practical Implications

Social media is receiving mounting attention in the health sector. However, much health promotion and education in social media is only designed for mass information dissemination [40] by promoting appropriate knowledge and attitudes. Therefore, social media in health promotion and education is one-way communication. However, health behavior is more complex and dynamic; knowledge and attitude are not sufficient to change behavior. Our study argues that child feeding practices are socially constructed, pointing out that mothers are notably motivated to follow group norms. Therefore, online communication programs should expand their functions of interactivity and collaboration among online members. While there is no “one size fits all” approach, our study highlighted some fundamental characteristics of potentially effective implementation of child feeding promotion in social media. First, an interactive process of exchange of information and support must be taken into account when designing a program. The aim is to facilitate conversations about the opinions and behavior of others to create or reinforce collective change in the targeted behavior.

Secondly, since mothers view the official guidelines as lacking practical application, it is essential to provide practical how-to tips and steps to supplement the guidelines. In the fast-paced modern lifestyle, many 21st century mothers may no longer have competent cooking skills. As modern mothers seek food recipes with appealing pictures that are endorsed by peers, online programs should promote healthy step-by-step recipes by using attractive photos incorporating social interactions by encouraging parents to give their comments and reviews. Additionally, these healthy recipes should be constructed with locally-sourced ingredients.

### 4.2. Limitations

The present findings should be interpreted in light of a few limitations. First, the participants were purposely recruited to address the study’s aim to explore why Thai mothers joined a Facebook group on children’s diets and their experiences. The participants had expertise in using the platform and were, therefore, ideally suited to providing insights into the social media’s online contents. Although the number of participants was small, they are fairly homogenous, making the sample size sufficient for this type of exploratory research [41]. The results may not be generalized to other online community platforms. The study participants were recruited from our previous survey; most of them had higher education levels and household incomes than the national average, but this is the group that are likely to be early adopters of social media [42]. Moreover, this study is based on maternal experiences on Facebook groups devoted to young children’s diets in general. The findings may be different among other Facebook peer support groups for more specific groups, such as for those with children with food allergies. Despite this limitation, to our knowledge, this is the first study in developing countries to explore the shift in maternal interest from traditional informational sources to Facebook support groups on children’s diets. Understanding why Thai mothers participated in online groups and how they appraise shared content will help public health professionals plan health promotion and communication in social media more effectively.

## 5. Conclusions

This interview study explored Thai millennial mothers’ experiences of Facebook child feeding support groups. It provides an insight into maternal perceptions and adoption of social connections and support in virtual communities. It also revealed factors that influenced how mothers select shared recipes for infants and young children. With the advantage of the social functions of online communities, health organizations could adopt these social processes to better harness their health promotion and communication on social media. Because of the potential influence on behavior, future research should investigate the effectiveness of social strategies and peer collaboration in social media on health promotion and communication programs.

## Data Availability

Not applicable.

## Appendix A

Interview guide:

1. How did you first come to find Facebook child feeding support groups? Prompt- What made you decide to join the group?
2. Tell me about your experiences joining Facebook child feeding support groups?
3. What can you get from the group that you cannot get from feeding guideline/parenting books/healthcare provider/family members?
4. How do you decide if you believe or trust other peers’ ideas/experiences/shared recipes on the group?
5. Have you asked group members for any help on feeding problem? Prompt-Have you adopted that advice? Why?
6. Have you shared your experiences/ideas/recipes around child feeding in the group? Prompt-Why?
